# Accelerating the Assessment of Hysteresis in Perovskite Solar Cells

**DOI:** 10.1021/acsenergylett.3c02779

**Published:** 2024-01-17

**Authors:** Enrique H. Balaguera, Juan Bisquert

**Affiliations:** † Escuela Superior de Ciencias Experimentales y Tecnología (ESCET), Universidad Rey Juan Carlos, 28933 Móstoles, Madrid, Spain; ‡ Institute of Advanced Materials (INAM), Universitat Jaume I, 12006 Castelló, Spain

## Abstract

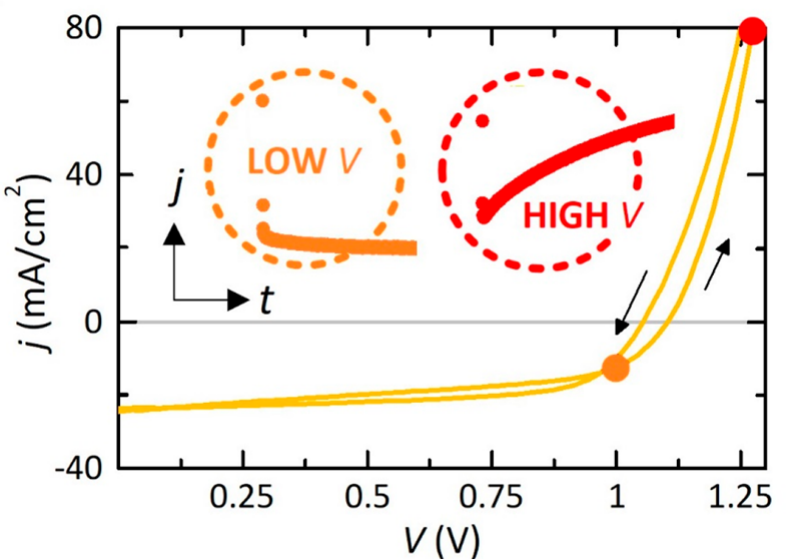

Halide perovskite materials have reached important milestones in the photovoltaic field, positioning them as realistic alternatives to conventional solar cells. However, unavoidable kinetic phenomena have represented a major concern for reliable steady-state performance assessment from standard current–voltage measurements. In particular, the dynamic hysteresis of current–voltage curves requires relatively long stabilization to achieve a credible figure for the power conversion efficiency. Hysteresis is caused by complex current transient phenomena that become active during staircase voltammetry. Here, we address the root of this problem. We pinpoint the dynamic characteristics behind the slow transient responses to strategically predict a minimum time delay and thus estimate the power conversion efficiency under steady-state conditions. Circuit-element analysis and impedance spectroscopy confirm our predictions based on an advanced transient study. Our results fundamentally explore the possibility of reducing data time acquisition in a reliable performance assessment, providing disruptive solutions and perspectives toward systematic production of photovoltaic perovskites.

To evaluate device performance in perovskite photovoltaics, it is important to understand the memory properties, based on internal ionic–electronic effects, of this semiconductor material.^1,2^ One of the most famous causes of these inherent perovskite effects is the anomalous hysteresis, present, since early studies, in the current–voltage curves.^3−6^ By interface engineering strategies^7,8^ and adequate measurement protocols,^9−12^ it has been possible to minimize the “hysteria around current-voltage hysteresis” in the research field.

Nevertheless, hysteresis mechanisms persist even in recent perovskite devices with high performance metrics.^13^ In order to circumvent the hysteresis problem,^14,15^ alternative techniques for assessing the performance of photovoltaic perovskites have been employed in the process of independent power conversion efficiency certification by cell calibration laboratories such as NREL or Newport (e.g., quasi steady-state current–voltage measurements and protocols based on stabilized power outputs near the maximum power point, MPP).^13,16^ However, these standardized but time-consuming practices can lead, in certain scenarios, to potential intrinsic device instabilities due to the use of *ad hoc* slow scan rates. Thus, the stability of the perovskites under the efficiency testing protocols based on current–voltage scanning represents a significant challenge for pushing this technology toward commercialization. In each current–voltage curve, the current is sampled in a discrete linear sense via staircase voltammetry. It follows that a reliable analysis of transient phenomenology in a stepwise voltage scanning, as indicator of charge-carrier dynamics in the transition between consecutive current recordings, can provide control over internal state variables and the respective relaxation effects that result in complex memory properties.^5,17,18^

In this Letter, we describe the multiple transient responses exhibited by perovskite solar cells along a current–voltage curve. As nonlinearity and memory effects play a dominant role in “hysteretical” current–voltage characteristics, we aim to determine a minimum delay time, in terms of scan rate, to carry out current–voltage measurements under steady-state conditions. Recently, hysteresis effects have been classified into two major types, capacitive and inductive, according to the dominant response of impedance spectroscopy.^19^ We use circuit theory as inspiration that describes the extraordinarily complex physical processes that turn out to be ubiquitous in halide perovskites, requiring eventual transformations of electrical elements along the voltage changes. Throughout our study, the multiple versions of transient responses exhibited in a halide perovskite that commonly undergo transformation from capacitive to inductive properties in a single current–voltage curve are introduced in accordance with the experimental observations reported in the literature. Analysis of our representative photovoltaic perovskites shows that the proposed numerical approximations are satisfied by the experimental data. Our procedure, easily implementable as an industrial-level automatic routine that monitors the real-time current during the current–voltage protocol, could continuously adapt the measurement conditions as the perovskite solar cells show important changes in the dynamic transient responses regarding device operation in the context of different degradation pathways.^20^

The representative structure of the photovoltaic perovskites in this study is fluorine-doped tin oxide (FTO)/compact titanium dioxide (c-TiO_2_, 30 nm)/mesoporous titanium dioxide (m-TiO_2_, 125 nm)/perovskite (350 nm)/2,2′,7,7′-Tetrakis[N,N-di(4-methoxyphenyl)amino]-9,9′-spiro-bifluorene (spiro-OMeTAD, 150 nm)/Au (100 nm), with a nominal active layer composition of Cs_0.05_Rb_0.05_MA_0.15_FA_0.75_Pb_1.05_(Br_0.05_I_0.95_)_3_. All fabrication procedures and electrical measurements are described in the Supporting Information. We obtained device performance metrics of around 20% derived from current–voltage measurements in the forward and reverse scan directions, which are within the typical values reported for this formulation.^21^ Nevertheless, as shown in Figure 1a, the estimate of the real efficiency is not trivial since considerable hysteresis mechanisms emerge, both normal and inverted. At this point, it is necessary to put the transition between discrete photocurrent values under a magnifying glass, thus requiring an “intelligent transient analysis” to accurately capture steady-state current–voltage responses. This approach is, however, perceived as highly complicated because it requires certain mathematical and engineering expertise from the perspective of nonlinear electrical circuits.^12^ Our representative metal halide perovskite, based on a mesoporous n-i-p configuration, shows different types of transient responses in separate voltage domains, as also reflected by literature results.^22,23^ From this closer look at the stepwise current–voltage measurements, we observe, after an initial faithful reproduction of the step transition, voltage-dependent gradual transient decays in the region of regular hysteresis for low applied voltages (see Figure 1b,c). In contrast, at high voltages, final abrupt rises in current dynamics are found in Figure 1d,e leading to the inverted hysteresis of the perovskite device. All these transient effects clearly underlie the physical mechanisms associated with electrical relaxation processes in the sense of transport, accumulation, and recombination of ionic and electronic charges.^24,25^ Therefore, the design of transient analysis protocols in current–voltage measurements to follow or, at least, to use as complementary information is essential to adhere to standard procedures and thus ensure that the reported perovskite solar cells have been obtained under steady-state conditions.

**Figure 1:**
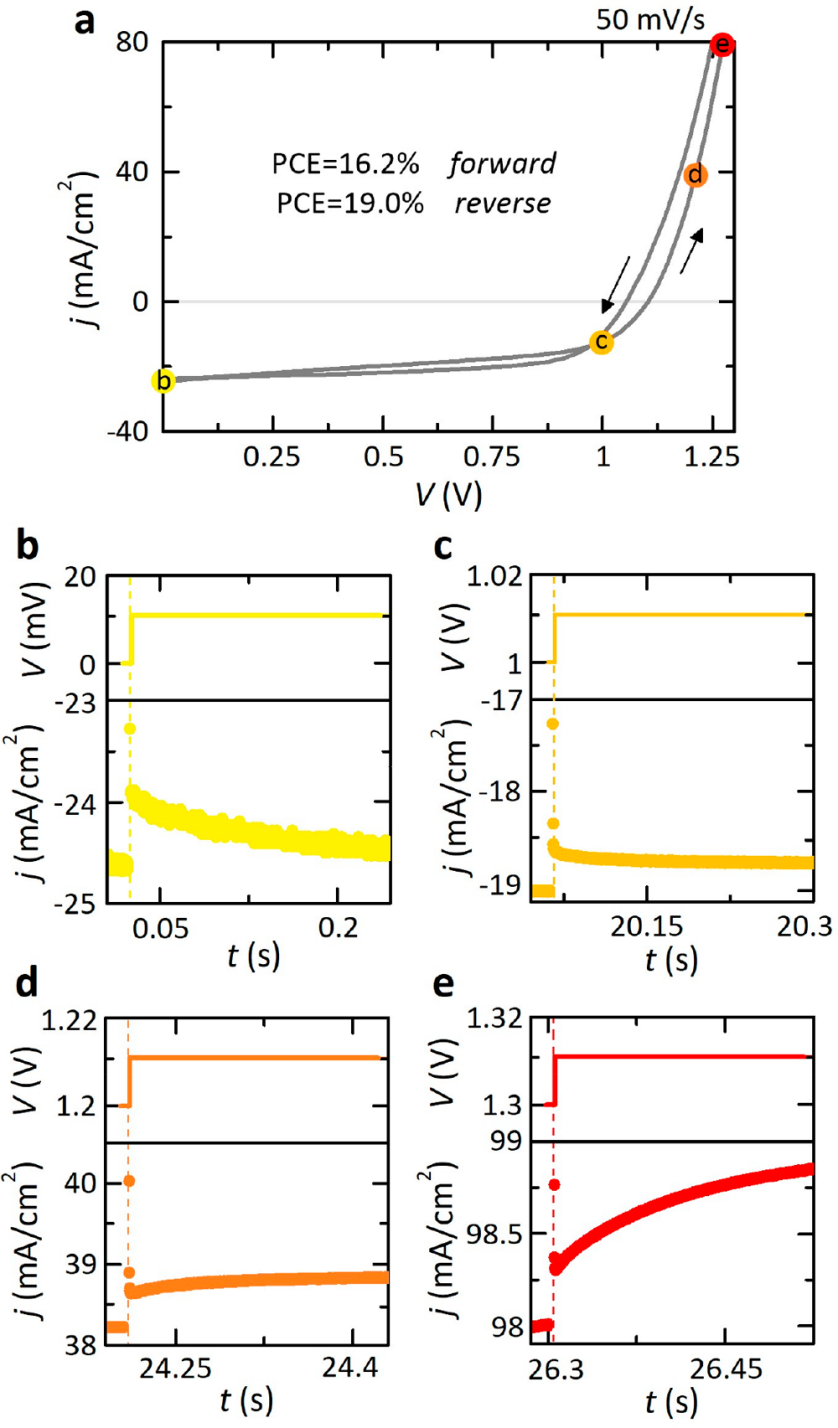
(a) Representative current–voltage curve of our quadruple-cation perovskite solar cell obtained using a step-size of 10 mV and a voltage scan rate of 50 mV/s. Halide perovskite exhibits normal and inverted hysteresis derived by the voltage-dependent transient dynamics of the photocurrent responses, consisting of decays at low voltages, (b) 0 V and (c) 1 V, and negative spike components with abrupt final rises in current for the high-voltage domain, (c) 1.2 V and (c) 1.3 V.

Based on the ionic–electronic properties of the perovskite semiconductors commonly manifested throughout a current–voltage curve in perovskite solar cells, we formulated a familiar dynamical model^26−28^ outlined primarily from the following expression:
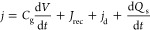
1where the total current flowing through the solar cell *j* is divided into four distinct pathways: (i) a displacement current that charges the geometrical capacitance *C*_g_ of the perovskite material; conduction channels in which the current may be extracted from the contacts (ii) instantaneously via recombination processes *J*_rec_ or (iii) slowly with an ion-modulated current *j*_d_; and (iv) an interfacial current in the sense of a corresponding charge *Q*_s_. For the recombination current, note that we consider, throughout the Letter, *J*_rec_ = *J*_rec0_*e*^*qV*/*n*_rec_*kT*^, where *q* is the electron charge, *n*_rec_ is an ideality factor, *k* is Boltzmann's constant, and *T* is the absolute temperature. This gives rise to the conductance *g*_rec_ = d*J*_rec_/d*V*. Nevertheless, the current *j*_d_ cannot follow the external voltage *V* rapidly, reacting slowly to achieve the steady-state conditions.^27,29^ On the other hand, the surface charge *Q*_s_, a function of an internal voltage *V*_s_ and thus introducing an interfacial capacitance *C*_s_ = d*Q*_s_/d*V*_s_, also shows a retarded dynamic due to ionic charges.^26,30^ In this way, the variables *V*_s_ and *j*_d_ generate the famous memory effects in perovskites with characteristic behaviors described, respectively, as
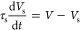
2
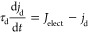
3that control the voltage-dependent conductivity in the perovskite materials depending on the ionic kinetics. In eq 2, the slow variable, for convenience, is the surface polarization voltage *V*_s_, governed by the characteristic time τ_s_, which introduces a transitory ionic conductance, *g*_ion_ = 1/τ_s_(d*Q*_s_/d*V*_s_) = *C*_s_/τ_s_.^26^ This relaxation process is commonly visible in the low-voltage region. On the other hand, the dynamical behavior of the current *j*_d_ in eq 3, dominant at high voltages, models the process that establishes an additional conductivity channel causing the memory effect by a slow recovery of relaxation time τ_d_ in response to the external stimulus *V*.^26,30^ In effect, metal halide perovskite devices commonly undergo a transformation in the dynamics of the slow current components, both related to ionic-controlled recombination processes via surface polarization effects.^12,27^ The kinetic properties at long time scales are modulated by slight variations of the characteristic time constants (τ_s_ and τ_d_) that determine the dominant relaxation response (eq 2 or 3) at different voltage regions. In eq 3, *J*_elect_ represents the stationary value of the delayed current that we again assume is governed by an exponential dependence, *J*_elect_ = *J*_elect0_*e*^*qV*/*n*_elect_*kT*^, based on the experimental observation in the literature.^31−34^ Thus, the additional conductivity pathway in equilibrium can be also modeled from *g*_elect_ = d*J*_elect_/d*V*. Two types of electronic conductances (*g*_rec_ and *g*_elect_) indeed emerge in the model with similar nature but exhibiting different physics beyond the time scales behind them. The first represents the immediate recombination of photogenerated charges. The second is related to an ionic reorganization in the device that enhances the electronic current in terms of charge collection or trapping. Importantly, this delayed photocurrent mode forms the prominent feature of the chemical inductor, *L*_d_ = τ_d_(d*J*_elect_/d*V*)^−1^ = τ_d_*g*_elect_. As mentioned in previous works,^35,36^ this inductive process is a general feature present in many processes and materials, caused by a delay effect on the electronic phenomena associated with slow ionic mechanisms and related to the negative capacitance. The idea behind the general term “chemical inductor” is that this electrical element is not based on electromagnetic effects but arises from mixed ionic–electronic conduction in complex materials. Therefore, the model has a total of two memory variables that interchange the governability of the response at long time scales in the current–voltage curves of perovskite solar cells due to the nonlinear ionic–electronic dynamic complexity of this material. However, the slow kinetic properties in perovskites, based on ionic-controlled surface recombination processes,^37−39^ are commonly regulated by a single relaxation time
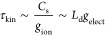
4due to the characteristic memory-based coupling of these materials whose phenomenological consequence is that τ_s_ ∼ τ_d_.^22,29,40^ In essence, this identification means that the slow current and surface polarization are both caused by a single variable, as reported experimentally.^27^ A similar framework analysis has been successfully used to explain the dynamic hysteresis^26^ and linear impedance patterns^29,30^ of perovskite solar cells. Here, we will try to explain the experimental transient dynamics observed during consecutive recordings in current–voltage measurements with the aim of establishing determined conditions to reach steady-state values and eliminate hysteresis.

According to our physical model, we consider the electrical representation of a perovskite solar cell, as shown in Figure 2a. Inspired by the multiple versions of current transient responses to stepwise voltage scanning observed in Figure 1, this advanced nonlinear electrical circuit models the voltage-dependent behavior in the time-domain along the current–voltage curve of halide perovskites, transforming dominant hysteretic dynamics from capacitive to inductive in nature as the bias point increases. This equivalent circuit, in addition to qualitatively explaining transient behavior in terms of conductances, helps to articulate a consistent interpretation of the complex physics behind this ionic–electronic conductor^29,30^ in comparison with other electrical models reported in the literature.^27,31^

**Figure 2:**
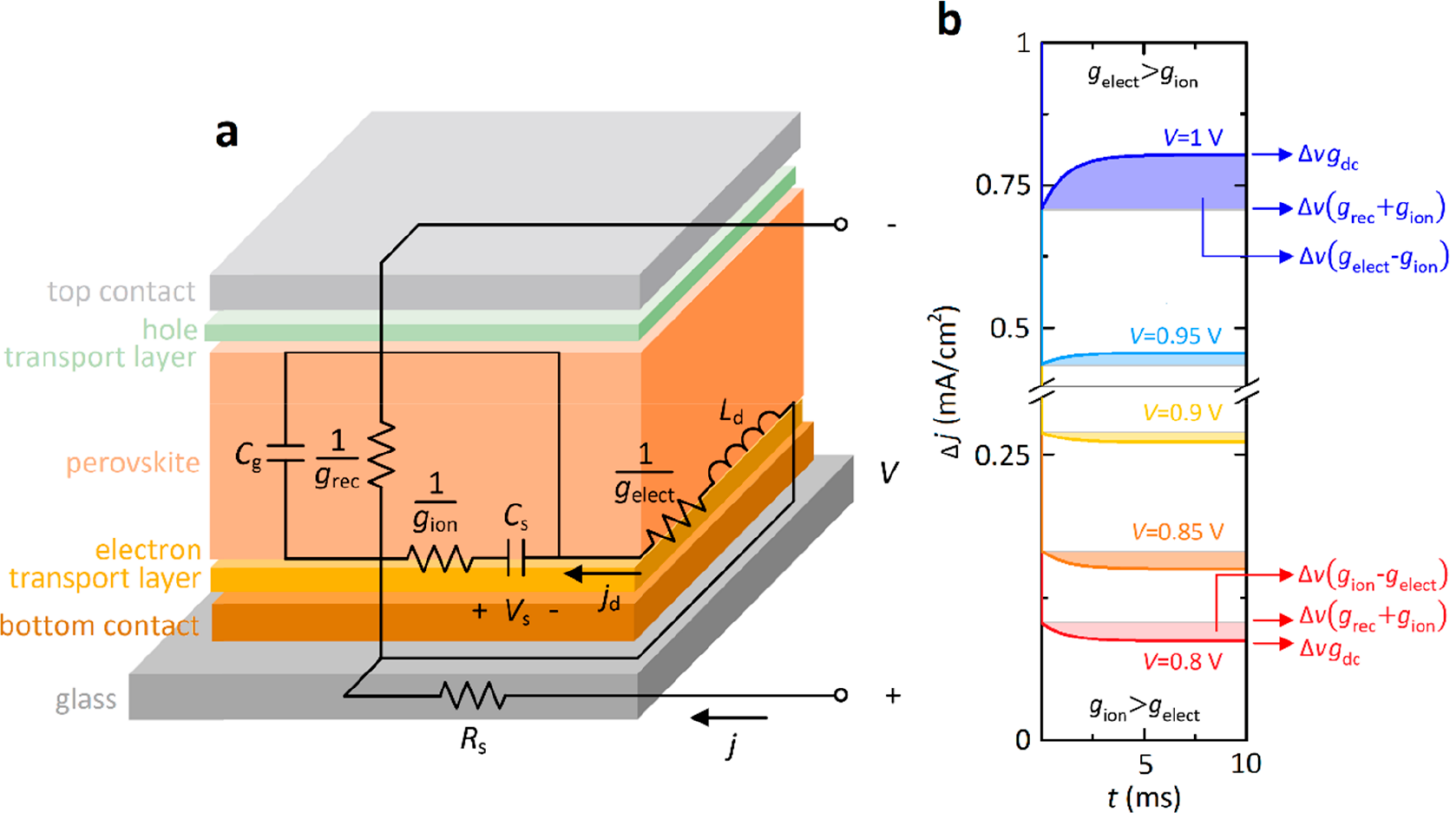
(a) Equivalent circuit derived from the transient responses and the schematic of the layer structure composing the perovskite device. (b) Set of time transients generated for Δ*v* = 10 mV, *R*_s_ = 10 Ω, τ_v_ = 1 μs, *g*_rec_ = 3*e*^*V*/0.11^ mS/cm^2^, *g*_elect_ = 0.2*e*^*V*/0.08^ mS/cm^2^, and *g*_ion_ = 2*e*^*V*/0.1^ mS/cm^2^, τ_kin_ = 1 ms, and voltage as indicated.

When one changes the applied potential in stepwise current–voltage measurements, the new equilibrium point totally modifies the transient response^12,17^ as, in effect, illustrated in Figure 1b–e. To analyze the temporal evolution at a given voltage bias under small-signal conditions, we obtain important insights into the transient dynamics from the linearized equations of our three-dimensional physical model (eqs 1–3) at a steady-state point. For a step change of applied potential Δ*v* in the context of stepwise current–voltage measurements, the resulting small-amplitude current response Δ*j*(*t*) in the time-domain can be determined, in terms of operational Δ*j*_ss_ and memory Δ*j*_mem_(*t*) contributions, from the following model equations:
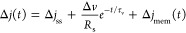
5
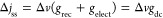
6
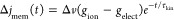
7obtained as the natural solution, via numerical integration methods or the Laplace transform technique, of the previous set of the differential equations (refer to eqs 1–3) in the linear version. The corresponding theoretical analysis is described in detail in the Supporting Information.

Although it is irrelevant in the destabilization process (hysteresis) of the current–voltage curves, we first describe the additional term in eq 5 as an ultrafast process corresponding to the charge of the geometrical capacitance, by a characteristic time of τ_v_ = *R*_s_*C*_g_.^41,42^ This capacitive current can be indeed found in all the transient responses of Figure 1, in the form of initial sharp jump discontinuities followed by ultrafast decays, due to the series resistance *R*_s_ and the charging of the constant capacitance *C*_g_ whose origin is purely bulk dielectric in nature. Note that we consider throughout the Letter, for simplicity, that the parasitic series resistance effects are negligible (*R*_s_ → 0) in comparison to the conductance states of the perovskite over the voltage range swept in the measurement under study.

Our model, with a clear physical meaning, shows that there are two recombination pathways in halide perovskites as reported in the literature: direct *g*_rec_ and delayed by ionic reorganization *g*_elect_,^28^ evidenced in dc conditions (refer to eq 6). The memory function Δ*j*_mem_(*t*), on the other hand, results in an exponential function with a prefactor consisting of a combination of conductances with an ionic and electronic nature (*g*_ion_ and *g*_elect_, respectively) and the characteristic relaxation time τ_kin_ that generalizes the electrical charge coupling in halide perovskites. In effect, eq 7 provides a theoretical visualization of the interplay between perovskites’ ionic and electronic responses in the appearance of current–voltage hysteresis.^43^ Following the rule of transient dynamics in Figures 1b–e, we obtain *g*_ion_ > *g*_elect_ at low voltages (gradual decays, *V* → 0) and *g*_ion_ < *g*_elect_ in the high-voltage region (abrupt rises in current at long time scales, *V* → *V*_oc_). To summarize, Figure 2b shows the graphical representation of simulated currents in the time domain varying the value of the applied voltage. Representative transient responses, with a dynamical behavior similar to those of Figure 1, have been labeled to reveal relevant memory-based currents in relation to our theory: Δ*v*(*g*_rec_ + *g*_ion_) and Δ*vg*_dc_ for *t* ≪ τ_kin_ and *t* ≫ τ_kin_, respectively, and the difference between both terms that results in the interesting value Δ*v*(*g*_ion_ – *g*_elect_). It is clearly seen that this last parameter decreases as the bias voltage increases, going from positive to negative values and thus inverting the transient dynamics. Importantly, the results obtained from numerical simulations displayed a good qualitative agreement with experimental data, giving a step beyond current–voltage curves and impedance spectra^25−30^ in modeling the rich phenomenology of perovskites by using this family of mathematical models.

Theoretically speaking, the necessary time delay for steady-state device operation, Δ*j*(*t*) → Δ*j*_ss_, and thus, to eliminate the memory traces (Δ*j*_mem_(Δ*t*_ss_) → 0) that give rise to the current–voltage hysteresis is that for which Δ*j*_ss_ ≫ Δ*j*_mem_(Δ*t*_ss_).^44^ We consider the 5% criterion, a typical tolerance band used in control engineering, yielding
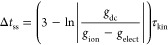
8In eq 8 appears the classical result 3τ_kin_ described in basic control engineering courses,^45^ but also an additional term originated by the ionic–electronic coupling of perovskite solar cells that modulates the conventional suitable time delay and makes emerge the discrepancy between forward and reverse scans when one simply selects the classical value. Thus, although the speed of transient dynamics depends fundamentally on the value of the time constant τ_kin_, the settling time Δ*t*_ss_ associated with steady-state conditions is also a function of a ratio of conductances.

The final step is the selection of the most restrictive voltage-dependent Δ*t*_ss_ in the large amplitude voltage scanning due to nonlinear characteristics of the perovskite devices. In effect, our physical model needs to be supplemented with the voltage dependence of the conductances. Assuming that the different conductances have approximately the same ideality factor (as in the numerical simulations of Figure 2b),^12,33,46^ we observe that Δ*t*_ss_ exhibits a voltage-independent behavior because τ_kin_ commonly remains constant along the voltage sweep (see above). Finally, the integration of the required time delay therefore gives the optimum scan rate to develop steady-state current–voltage measurements in perovskites solar cells:
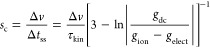
9stabilizing all the transient responses with multiple versions, due to their different origins, that constitute the current–voltage curves in halide perovskites, with the best overall compromise between non-time-consuming experiments, intrinsic device stability, and reliable estimation of the real device performance.

The time constant τ_kin_ of each time transient is plotted in Figure 3 as a function of voltage. We observe, in effect, that the applied voltage has not a considerable effect on the characteristic relaxation times, as remarked by García-Belmonte and co-workers.^46^ Furthermore, we point out that the relaxation time remains at approximately 75 ms even though a transition in the slow dominant mechanism occurs, from capacitive to inductive. This result justifies the identification of relaxation times presented in eq 4 (if the times are physically very different, a jump of *τ*_kin_ would be observed in Figure 3). Now, the corresponding time delays Δ*t*_ss_ are calculated via the model parameters extracted from the fitting of experimental time transients and are also presented in Figure 3. As we expected, we found an approximately voltage-independent value in the range of 2 s. This optimum time delay correlates to a value of scan rate of 5 mV/s considering a 10-mV of voltage step size from eq 9.

**Figure 3:**
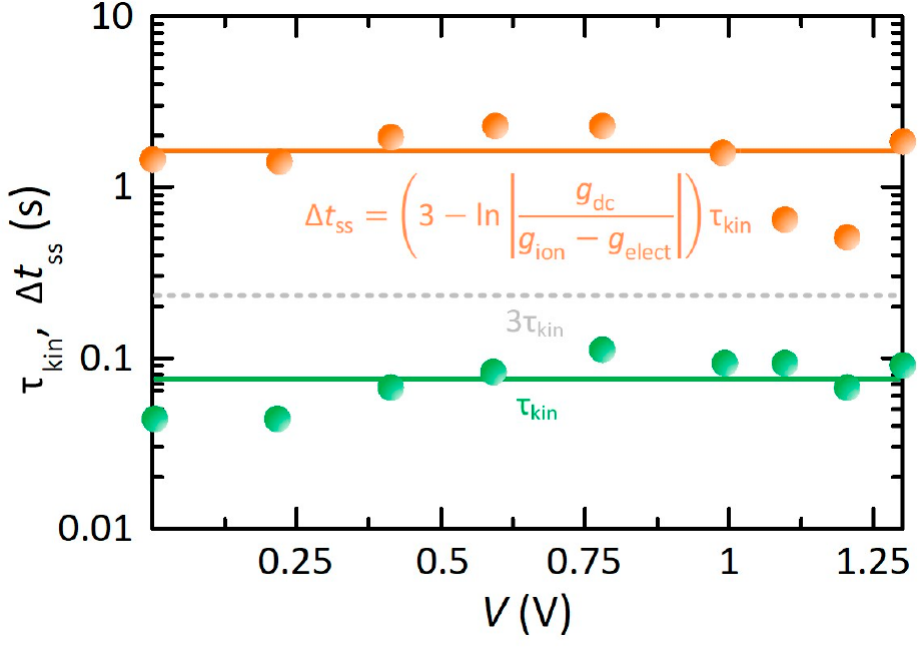
Time constants and optimum time delay extracted from the transient responses throughout the voltage range. In both characteristic times, the values remain independent of voltage at around 75 ms and 2 s for τ_kin_ and Δ*t*_ss_, respectively. Note that Δ*t*_ss_ is far from the classical value of 3τ_kin_.

To finalize from the general landscape of the experimental requirements criteria to stabilize the current–voltage curves in perovskite solar cells, we show a batch of curves at different voltage sweep rates in Figure 4. We represent the observed hysteresis traces, transforming the dominant behavior from normal (Figure 4a–c) to inverted (Figure 4d,e) as the scan rate decreases. The current–voltage curve is stabilized when the time transients reach equilibrium along the stepwise scanning at the hold time values Δ*t*_ss_ in decreasing sweep rates determined from transient analysis, as illustrated in Figure 4f. In effect, if one uses larger values for the steady-state time selected without any basis, one will also obtain stabilized current–voltage curves. However, this scenario would accelerate degradation pathways in the perovskite solar cells, derived from a long-term evaluation of devices,^47^ e.g., the measurement time of our current–voltage curve with a scan rate of 5 mV/s is 9 min, being 43 min if the sweep speed is 1 mV/s. The reproducibility of our methodology was checked and assured by conducting the experimental measurements in 12 samples with the same device configuration. Furthermore, we also present experimental evidence that confirms the validity of the control strategy of hysteresis presented here in planar p-i-n photovoltaic perovskites in the Supporting Information.

**Figure 4:**
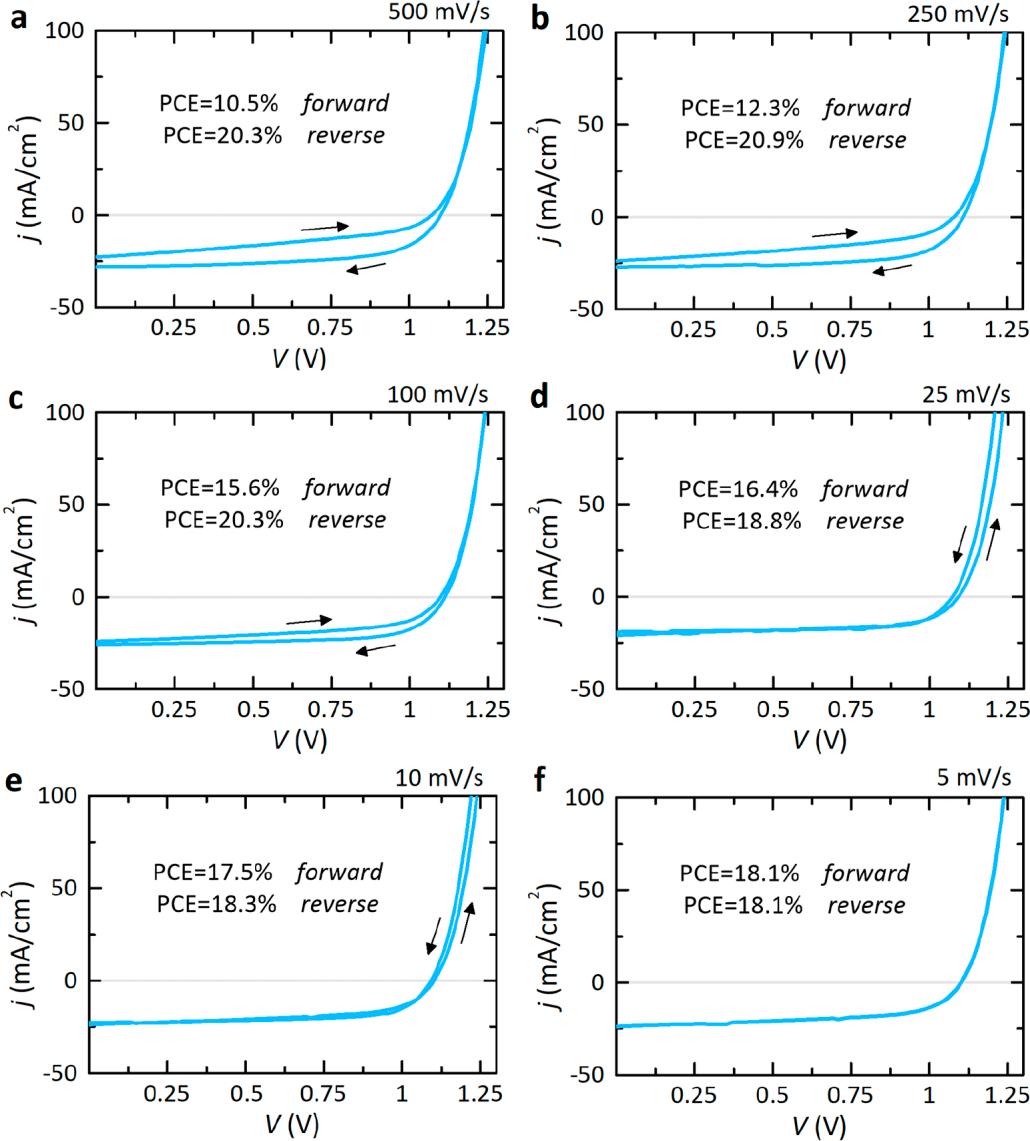
Catalogue of measured current–voltage responses for our perovskite solar cell as a function of the scan rate: (a) 500, (b) 250, (c) 100, (d) 25, (e) 10, and (f) 5 mV/s. Hysteresis mechanisms are, in effect, eliminated around the value of time delay and scan rate estimated here.

Taking into account the convenient equivalent circuit interpretation of the model, we can safely assume that impedance measurements are an alternative method to extract the dynamic operating parameters, with the aid of the interpretative potential of this technique.^31,48,49^ Therefore, such measurements were performed, although we will represent the experimental data in the form of *admittance responses* to better display the correlation of the conductances and time constants with the current–voltage experiments.

Figure 5 shows the evolution of the characteristic admittance patterns of perovskite solar cells, along the different points of the current–voltage curve, by representing the measured points in complex plane plots with the implicit frequency clockwise increasing. The spectral pictures, at low voltages, show two capacitive semicircles that provide information on the recombination kinetics in the bulk and the contacts. As one increases the voltage bias, the size of the arcs strongly decreases and a new feature, in the low-frequency region, emerges in the form of the chemical inductor (the famous negative capacitance), related to the accumulation of ions followed by ion-induced recombination, that increases dc conductance.^22,50−52^ For the interpretation of impedance spectroscopy of our experimental results, we develop again our three-dimensional model (eqs 1–3) into the small-perturbation equations; we subsequently take the Laplace transform in terms of the variable *s = j*ω where  (do not confuse with current density), and we finally calculate the admittance *Y*(*j*ω) = *ĵ*/*V̂* that gives the result
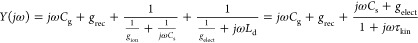
10where the “hysteretical relaxation processes” are controlled by the kinetic time constant τ_kin_ (pole of the transfer function, see eq 4). Note that the circumflex accent indicates small perturbation quantities. The admittance in eq 10 becomes that of the equivalent circuit of Figure 2a, with the addition of the series resistance *R*_s_. Further details on how to obtain the admittance function and the equivalent impedance plots of Figure 5 can be found in the Supporting Information. Beyond this visual inspection of the spectral features, we carried out a quantitative analysis to identify the value of the electrical parameters that determine the value of the steady-state time (see eqs 8 and 9). In effect, the procedure for the analysis of the admittance spectra is the same as that of impedance plots, requiring a fitting of the experimental results with an equivalent model (here, the electrical circuit of Figure 2a). However, the limiting behaviors of the admittance now are expressed in terms of conductances, and even more importantly, the characteristic frequencies are obtained as d*Y*_j_/dω = 0 (ω_HF_ and ω_LF_), exhibiting different values for the relaxation times of the impedance response. Graphically, time constants correspond to the inverse of the characteristic frequencies found from the maximum magnitudes of the imaginary part of the admittance in the resulting arcs (τ_v_ = 1/ω_HF_ and τ_kin_ = 1/ω_LF_), mathematically correlated with those of the current transient responses.^53^ From mathematical operations, we obtain again constant values of Δ*t*_ss_ ≈ 2 s and *s*_c_ ≈ 5 mV/s to reach equilibrium in all the transient responses along the stepwise current–voltage measurements.

**Figure 5:**
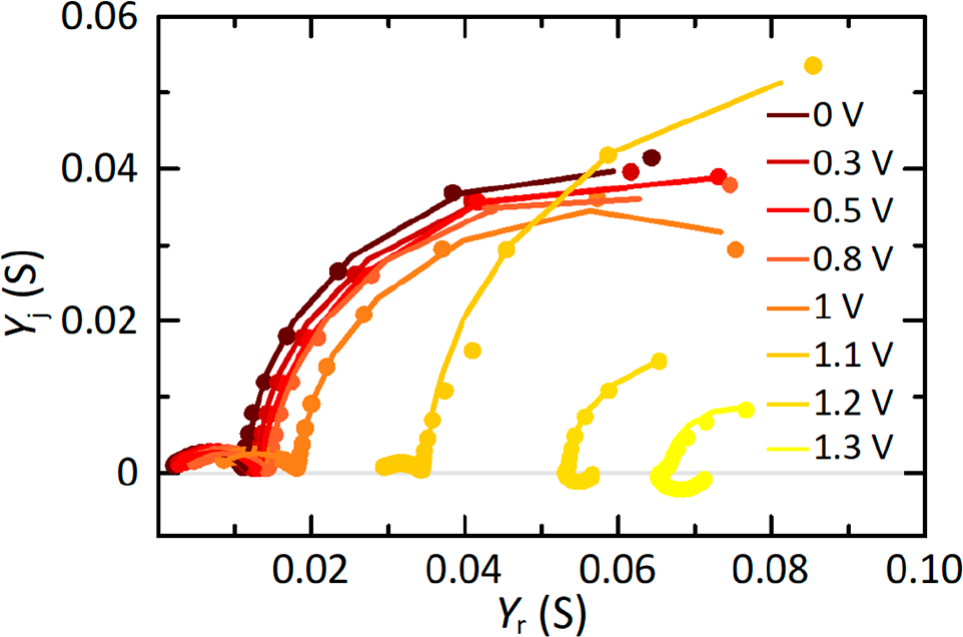
Admittance spectra patterns measured at different bias voltages to help model and interpret mathematically equivalent steady-state photocurrent transient responses. *Y*_r_ and *Y*_j_ are the real and imaginary parts of the admittance function, respectively.

Our procedure represents an alternative approach to the conventional efficiency testing practices based on conducting stable power outputs over a range of bias voltages that cover the MPP.^54−56^ One of the most important drawbacks of this type of measurement is the time consumption of the order of ten minutes (equally distributed to extract the kinetic parameters and apply the MPP tracking),^57^ leading to a continuous demand of optimized protocols that circumvent the recurrent problem of time-variance in metal halide perovskites due to reversible and irreversible degradation during the performance assessment.^58,59^ The essential step here is to achieve a significant reduction in the data acquisition time with reliable precision in the cell efficiency measurement in comparison to the classical protocol commonly used by experimentalists, maximizing in addition the information about the electrical behavior of photovoltaic devices and retaining the advantages of classical current–voltage measurements.

To provide a guideline to experimentalists, we explain the measurement protocol proposed that, in summary, is as outlined in Figure 6: (i) It starts with an introductory fast current–voltage curve to explore the level of hysteresis. Thereafter, (ii) one has to carry out small perturbation choroamperometric experiments at low and high voltages to determine if there exists a transformation in the slow transient dynamics. An alternative approach to small-amplitude time-domain measurements consists of implementing the equivalent technique of impedance spectroscopy to obtain the value of the corresponding conductances and time constants that determine the value of Δ*t*_ss_ and *s*_c_. Note that impedance measurements have been already correlated with the hysteresis level in current–voltage curves.^12,41,58^ Then, (iii) one must extract the optimal scan rate from the fitting of experimental time-domain (admittance) responses to eqs 5–7 (eq 10) in order to find hysteresis-free current–voltage curves with the aid of eqs 8 and 9. Similar values were obtained from both responses (eq 4). Once the stabilization of the transient responses has been guaranteed, (iv) the current–voltage measurement under the voltage sweep velocity determined in (iii) can be carried out in both forward and reverse directions (for verification purposes) to finally determine the power conversion efficiency from light to electricity of the perovskite solar cell. In effect, there is a significant reduction in the measurement time when using this optimal protocol, decreasing by at least half—a few seconds in (i), in the order of microseconds in (ii), and a few hundred seconds (always less than 300 s) in (iv) applying a single sweep direction—to obtain the electrical responses.

**Figure 6:**
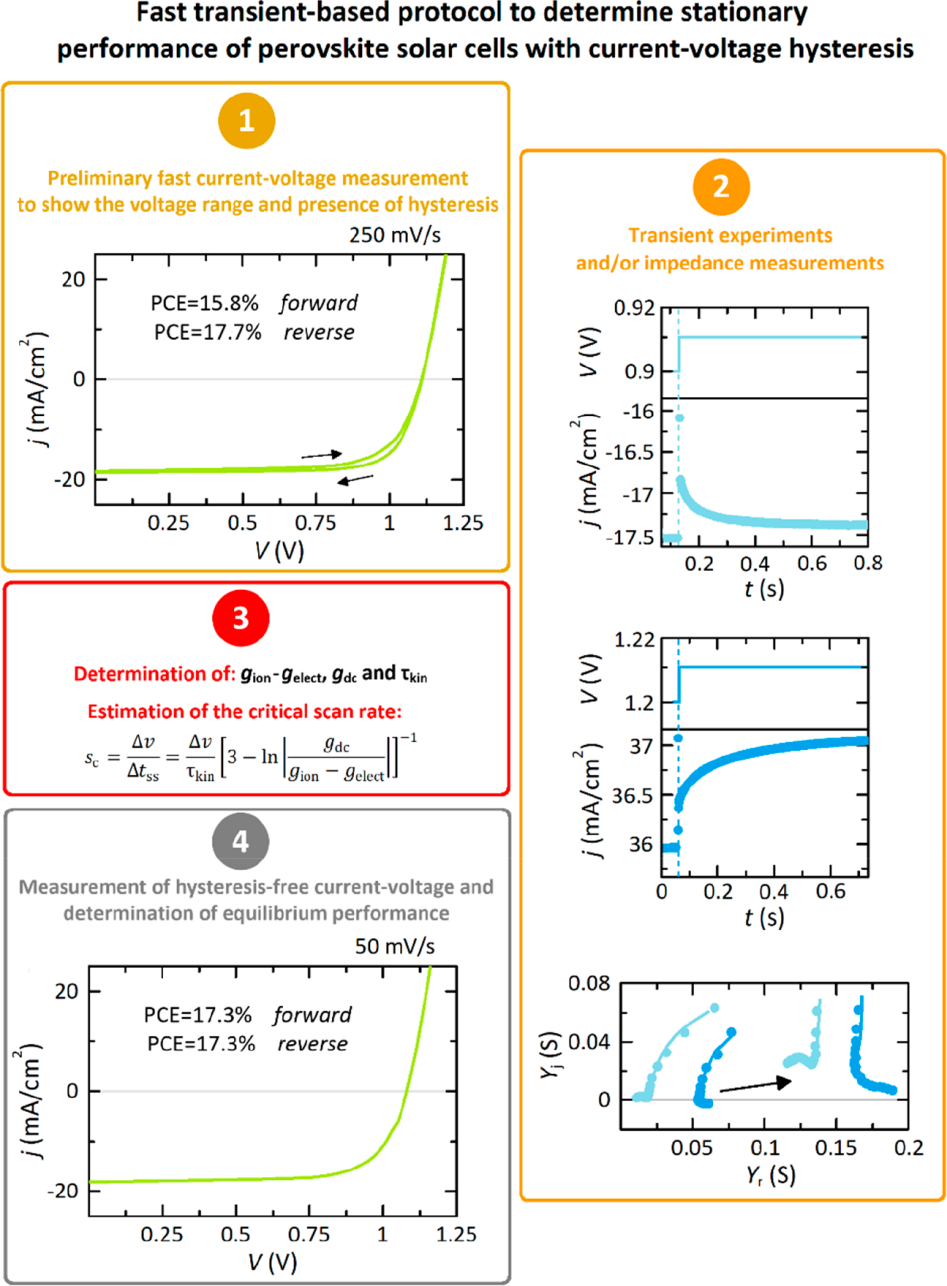
Fast experimental methodology to determine the stationary performance of perovskite solar cells with hysteresis effects. The experimental data correspond to an inverted or p-i-n perovskite device, described in detail in the Supporting Information.

Standard testing protocols are of particular interest for assessing the solar energy conversion of photovoltaic perovskites in an industrialization phase due to the intense debate that remains around current–voltage hysteresis (normal and inverted) at the research level since the first reports of these electronic devices. In this context, we propose here a comprehensive exploration of stepwise time transient effects rooted in hysteresis mechanisms. By an exhaustive analysis of the discrete nature of the classical current–voltage curve in perovskite solar cells, we unveiled the intricate nature of slow transient responses (capacitive or inductive) that introduce memory traces under non-steady-state conditions. Through an analytical methodology that monitors the real-time current during current–voltage measurements, we delineated a suitable time delay to stabilize all the transient responses throughout the nonlinear curve without reducing the operational lifetime of perovskite devices due to unnecessary long-time experiments due to the use of *ad hoc* slow scan rates. Our results predict experimentally the dynamic behavior of memory modes in perovskite semiconductors, enabling rapid and massive characterization of high-performance cells by automated methods.

## Supporting Information Available

Theoretical analysis of the model in time- and frequency-domain; impedance spectra fitting results; application of the hysteresis control strategy in inverted perovskite devices; experimental section (PDF)

## References

[ref1] Eames C.; Frost J. M.; Barnes P. R. F.; O’Regan B. C.; Walsh A.; Islam M. S. Ionic Transport in Hybrid Lead Iodide Perovskite Solar Cells. Nat. Commun. 2015, 6 (1), 7497 10.1038/ncomms8497. 26105623 PMC4491179

[ref2] Azpiroz J. M.; Mosconi E.; Bisquert J.; De Angelis F. Defect Migration in Methylammonium Lead Iodide and Its Role in Perovskite Solar Cell Operation. Energy Environ. Sci. 2015, 8 (7), 2118–2127. 10.1039/C5EE01265A.

[ref3] Snaith H. J.; Abate A.; Ball J. M.; Eperon G. E.; Leijtens T.; Noel N. K.; Stranks S. D.; Wang J. T.-W.; Wojciechowski K.; Zhang W. Anomalous Hysteresis in Perovskite Solar Cells. J. Phys. Chem. Lett. 2014, 5, 1511–1515. 10.1021/jz500113x. 26270088

[ref4] Kim H.-S.; Park N.-G. Parameters Affecting I–V Hysteresis of CH_3_NH_3_PbI_3_ Perovskite Solar Cells: Effects of Perovskite Crystal Size and Mesoporous TiO_2_ Layer. J. Phys. Chem. Lett. 2014, 5, 2927–2934. 10.1021/jz501392m. 26278238

[ref5] Unger E. L.; Hoke E. T.; Bailie C. D.; Nguyen W. H.; Bowring A. R.; Heumüller T.; Christoforo M. G.; McGehee M. D. Hysteresis and transient behavior in current–voltage measurements of hybrid-perovskite absorber solar cells. Energy Environ. Sci. 2014, 7, 3690–3698. 10.1039/C4EE02465F.

[ref6] Tress W.; Marinova N.; Moehl T.; Zakeeruddin S. M.; Nazeeruddin M. K.; Gratzel M. Understanding the rate-dependent J-V hysteresis, slow time component, and aging in CH_3_NH_3_PbI_3_ perovskite solar cells: the role of a compensated electric field. Energy Environ. Sci. 2015, 8, 995–1004. 10.1039/C4EE03664F.

[ref7] Shao Y.; Xiao Z.; Bi C.; Yuan Y.; Huang J. Origin and elimination of photocurrent hysteresis by fullerene passivation in CH_3_NH_3_PbI_3_ planar heterojunction solar cells. Nat. Commun. 2014, 5, 5784 10.1038/ncomms6784. 25503258

[ref8] Yang D.; Zhou X.; Yang R.; Yang Z.; Yu W.; Wang X.; Li C.; Liu S.; Chang R. P. H. Surface optimization to eliminate hysteresis for record efficiency planar perovskite solar cells. Energy Environ. Sci. 2016, 9, 3071–3078. 10.1039/C6EE02139E.

[ref9] van Reenen S.; Kemerink M.; Snaith H. J. Modeling Anomalous Hysteresis in Perovskite Solar Cells. J. Phys. Chem. Lett. 2015, 6, 3808–3814. 10.1021/acs.jpclett.5b01645. 26722875

[ref10] Kim H.-S.; Jang I.-H.; Ahn N.; Choi M.; Guerrero A.; Bisquert J.; Park N.-G. Control of *I*–*V* Hysteresis in CH_3_NH_3_PbI_3_ Perovskite Solar Cell. J. Phys. Chem. Lett. 2015, 6 (22), 4633–4639. 10.1021/acs.jpclett.5b02273. 26551249

[ref11] Rong Y. G.; Hu Y.; Ravishankar S.; Liu H. W.; Hou X. M.; Sheng Y. S.; Mei A. Y.; Wang Q. F.; Li D. Y.; Xu M.; et al. Tunable hysteresis effect for perovskite solar cells. Energy Environ. Sci. 2017, 10, 2383–2391. 10.1039/C7EE02048A.

[ref12] Bisquert J.; Guerrero A.; Gonzales C. Theory of Hysteresis in Halide Perovskites by Integration of the Equivalent Circuit. ACS Phys. Chem. Au 2021, 1 (1), 25–44. 10.1021/acsphyschemau.1c00009. 36855663 PMC9718316

[ref13] Jeong J.; Kim M.; Seo J.; Lu H.; Ahlawat P.; Mishra A.; Yang Y.; Hope M. A.; Eickemeyer F. T.; Kim M.; et al. Pseudo-halide anion engineering for α-FAPbI_3_ perovskite solar cells. Nature 2021, 592, 381–385. 10.1038/s41586-021-03406-5. 33820983

[ref14] Almora O.; Aranda C.; Zarazua I.; Guerrero A.; Garcia-Belmonte G. Noncapacitive Hysteresis in Perovskite Solar Cells at Room Temperature. ACS Energy Lett. 2016, 1, 209–215. 10.1021/acsenergylett.6b00116.

[ref15] Tress W.; Baena J. P. C.; Saliba M.; Abate A.; Graetzel M. Inverted Current–Voltage Hysteresis in Mixed Perovskite Solar Cells: Polarization, Energy Barriers, and Defect Recombination. Adv. Energy Mater. 2016, 6 (19), 1600396 10.1002/aenm.201600396.

[ref16] Kim G.-H.; Kim D. S. Development of perovskite solar cells with > 25% conversion efficiency. Joule 2021, 5, 1033–1035. 10.1016/j.joule.2021.04.008.

[ref17] Chen B.; Yang M.; Zheng X.; Wu C.; Li W.; Yan Y.; Bisquert J.; Garcia-Belmonte G.; Zhu K.; Priya S. Impact of capacitive effect and ion migration on the hysteretic behavior of perovskite solar cells. J. Phys. Chem. Lett. 2015, 6, 4693–4700. 10.1021/acs.jpclett.5b02229. 26550850

[ref18] Hill N. S.; Cowley M. V.; Gluck N.; Fsadni M. H.; Clarke W.; Hu Y.; Wolf M. J.; Healy N.; Freitag M.; Penfold T. J.; et al. Ionic Accumulation as a Diagnostic Tool in Perovskite Solar Cells: Characterizing Band Alignment with Rapid Voltage Pulses. Adv. Mater. 2023, 35, 2302146 10.1002/adma.202302146. 37145114

[ref19] Bisquert J. Inductive and Capacitive Hysteresis of Current-Voltage Curves: Unified Structural Dynamics in Solar Energy Devices, Memristors, Ionic Transistors, and Bioelectronics. PRX Energy 2024, 3, 011001 10.1103/PRXEnergy.3.011001.

[ref20] Hartono N. T. P.; Köbler H.; Graniero P.; Khenkin M.; Schlatmann R.; Ulbrich C.; Abate A. Stability follows efficiency based on the analysis of a large perovskite solar cells ageing dataset. Nat. Commun. 2023, 14, 4869 10.1038/s41467-023-40585-3. 37573324 PMC10423264

[ref21] Xie H.; Wang Z.; Chen Z.; Pereyra C.; Pols M.; Gałkowski K.; Anaya M.; Fu S.; Jia X.; Tang P.; et al. Decoupling the Effects of Defects on Efficiency and Stability through Phosphonates in Stable Halide Perovskite Solar Cells. Joule 2021, 5 (5), 1246–1266. 10.1016/j.joule.2021.04.003.

[ref22] Ebadi F.; Taghavinia N.; Mohammadpour R.; Hagfeldt A.; Tress W. Origin of Apparent Light-Enhanced and Negative Capacitance in Perovskite Solar Cells. Nat. Commun. 2019, 10 (1), 1574 10.1038/s41467-019-09079-z. 30952882 PMC6450882

[ref23] Hernández-Balaguera E.; Bisquert J. Negative Transient Spikes in Halide Perovskites. ACS Energy Lett. 2022, 7, 2602–2610. 10.1021/acsenergylett.2c01252.

[ref24] Jacobs D. A.; Wu Y.; Shen H.; Barugkin C.; Beck F. J.; White T. P.; Weber K.; Catchpole K. R. Hysteresis phenomena in perovskite solar cells: the many and varied effects of ionic accumulation. Phys. Chem. Chem. Phys. 2017, 19, 3094–3103. 10.1039/C6CP06989D. 28079207

[ref25] Moia D.; Gelmetti I.; Calado P.; Fisher W.; Stringer M.; Game O.; Hu Y.; Docampo P.; Lidzey D.; Palomares E.; et al. Ionic-to-Electronic Current Amplification in Hybrid Perovskite Solar Cells: Ionically Gated Transistor-Interface Circuit Model Explains Hysteresis and Impedance of Mixed Conducting Devices. Energy Environ. Sci. 2019, 12 (4), 1296–1308. 10.1039/C8EE02362J.

[ref26] Ravishankar S.; Almora O.; Echeverría-Arrondo C.; Ghahremanirad E.; Aranda C.; Guerrero A.; Fabregat-Santiago F.; Zaban A.; Garcia-Belmonte G.; Bisquert J. Surface Polarization Model for the Dynamic Hysteresis of Perovskite Solar Cells. J. Phys. Chem. Lett. 2017, 8 (5), 915–921. 10.1021/acs.jpclett.7b00045. 28170275

[ref27] Gonzales C.; Guerrero A.; Bisquert J. Transition from Capacitive to Inductive Hysteresis: A Neuron-Style Model to Correlate I–V Curves to Impedances of Metal Halide Perovskites. J. Phys. Chem. C 2022, 126, 13560–13578. 10.1021/acs.jpcc.2c02729.

[ref28] Filipoiu N.; Preda A. T.; Anghel D.-V.; Patru R.; Brophy R. E.; Kateb M.; Besleaga C.; Tomulescu A. G.; Pintilie I.; Manolescu A.; et al. Capacitive and Inductive Effects in Perovskite Solar Cells: The Different Roles of Ionic Current and Ionic Charge Accumulation. Phys. Rev. Appl. 2022, 18, 064087 10.1103/PhysRevApplied.18.064087.

[ref29] Bisquert J. Electrical Charge Coupling Dominates the Hysteresis Effect of Halide Perovskite Devices. J. Phys. Chem. Lett. 2023, 14, 1014–1021. 10.1021/acs.jpclett.2c03812. 36693135 PMC10883608

[ref30] Ghahremanirad E.; Bou A.; Olyaee S.; Bisquert J. Inductive Loop in the Impedance Response of Perovskite Solar Cells Explained by Surface Polarization Model. J. Phys. Chem. Lett. 2017, 8 (7), 1402–1406. 10.1021/acs.jpclett.7b00415. 28287736

[ref31] Guerrero A.; Bisquert J.; Garcia-Belmonte G. Impedance Spectroscopy of Metal Halide Perovskite Solar Cells from the Perspective of Equivalent Circuits. Chem. Rev. 2021, 121 (23), 14430–14484. 10.1021/acs.chemrev.1c00214. 34845904

[ref32] Caprioglio P.; Wolff C. M.; Sandberg O. J.; Armin A.; Rech B.; Albrecht S.; Neher D.; Stolterfoht M. On the Origin of the Ideality Factor in Perovskite Solar Cells. Adv. Energy Mater. 2020, 10, 2000502 10.1002/aenm.202000502.

[ref33] Almora O.; Cho K. T.; Aghazada S.; Zimmermann I.; Matt G. J.; Brabec C. J.; Nazeeruddin M. K.; Garcia-Belmonte G. Discerning recombination mechanisms and ideality factors through impedance analysis of high-efficiency perovskite solar cells. Nano Energy 2018, 48, 63–72. 10.1016/j.nanoen.2018.03.042.

[ref34] Bennett L. J.; Riquelme A. J.; Anta J. A.; Courtier N. E.; Richardson G. Avoiding Ionic Interference in Computing the Ideality Factor for Perovskite Solar Cells and an Analytical Theory of Their Impedance-Spectroscopy Response. Phys. Rev. Appl. 2023, 19, 014061 10.1103/PhysRevApplied.19.014061.

[ref35] Bisquert J.; Guerrero A. Chemical Inductor. J. Am. Chem. Soc. 2022, 144 (13), 5996–6009. 10.1021/jacs.2c00777. 35316040 PMC8991013

[ref36] Hernández-Balaguera E.; Arredondo B.; Pereyra C.; Lira-Cantú M. Parameterization of the apparent chemical inductance of metal halide perovskite solar cells exhibiting constant-phase-element behavior. J. Power Sources 2023, 560, 232614 10.1016/j.jpowsour.2022.232614.

[ref37] Sanchez R. S.; Gonzalez-Pedro V.; Lee J.-W.; Park N.-G.; Kang Y. S.; Mora-Sero I.; Bisquert J. Slow Dynamic Processes in Lead Halide Perovskite Solar Cells. Characteristic Times and Hysteresis. J. Phys. Chem. Lett. 2014, 5 (13), 2357–2363. 10.1021/jz5011187. 26279559

[ref38] Richardson G.; O’Kane S. E. J.; Niemann R. G.; Peltola T. A.; Foster J. M.; Cameron P. J.; Walker A. B. Can Slow-Moving Ions Explain Hysteresis in the Current–Voltage Curves of Perovskite Solar Cells?. Energy Environ. Sci. 2016, 9 (4), 1476–1485. 10.1039/C5EE02740C.

[ref39] Wang H.; Guerrero A.; Bou A.; Al-Mayouf A. M.; Bisquert J. Kinetic and material properties of interfaces governing slow response and long timescale phenomena in perovskite solar cells. Energy Environ. Sci. 2019, 12, 2054–2079. 10.1039/C9EE00802K.

[ref40] Hernández-Balaguera E.; Martín-Martín D. A Unified Description of the Electrical Properties with Complex Dynamical Patterns in Metal Halide Perovskite Photovoltaics. Fractal Fract. 2023, 7, 516 10.3390/fractalfract7070516.

[ref41] Hernández-Balaguera E.; Romero B.; Arredondo B.; del Pozo G.; Najafi M.; Galagan Y. The Dominant Role of Memory-Based Capacitive Hysteretic Currents in Operation of Photovoltaic Perovskites. Nano Energy 2020, 78, 105398 10.1016/j.nanoen.2020.105398.

[ref42] Hernández-Balaguera E.; del Pozo G.; Arredondo B.; Romero B.; Pereyra C.; Xie H.; Lira-Cantú M. Unraveling the Key Relationship Between Perovskite Capacitive Memory, Long Timescale Cooperative Relaxation Phenomena, and Anomalous *J*–*V* Hysteresis. Solar RRL 2021, 5 (4), 2000707 10.1002/solr.202000707.

[ref43] Calado P.; Telford A. M.; Bryant D.; Li X.; Nelson J.; O’Regan B. C.; Barnes P. R. F. Evidence for Ion Migration in Hybrid Perovskite Solar Cells with Minimal Hysteresis. Nat. Commun. 2016, 7, 13831 10.1038/ncomms13831. 28004653 PMC5192183

[ref44] Hernández-Balaguera E.; Muñoz-Díaz L.; Pereyra C.; Lira-Cantú M.; Najafi M.; Galagan Y. Universal control strategy for anomalous ionic-electronic phenomenology in perovskite solar cells efficiency measurements. Mater. Today Energy 2022, 27, 101031 10.1016/j.mtener.2022.101031.

[ref45] Ogata K. Modern Control Engineering; Prentice Hall, 2010.

[ref46] Zarazua I.; Han G.; Boix P. P.; Mhaisalkar S.; Fabregat-Santiago F.; Mora-Seró I.; Bisquert J.; Garcia-Belmonte G. Surface recombination and collection efficiency in perovskite solar cells from impedance analysis. J. Phys. Chem. Lett. 2016, 7, 5105–5113. 10.1021/acs.jpclett.6b02193. 27973858

[ref47] Khenkin M. V.; Katz E. A.; Abate A.; Bardizza G.; Berry J. J.; Brabec C.; Brunetti F.; Bulović V.; Burlingame Q.; Di Carlo A.; et al. Consensus statement for stability assessment and reporting for perovskite photovoltaics based on ISOS procedures. Nature Energy 2020, 5, 35–49. 10.1038/s41560-019-0529-5.

[ref48] López-Varo P.; Jiménez-Tejada J. A.; García-Rosell M.; Ravishankar S.; Garcia-Belmonte G.; Bisquert J.; Almora O. Device physics of hybrid perovskite solar cells: theory and experiment. Adv. Energy Mater. 2018, 8, 1702772 10.1002/aenm.201702772.

[ref49] von Hauff E.; Klotz D. Impedance spectroscopy for perovskite solar cells: characterisation, analysis, and diagnosis. J. Mater. Chem. C 2022, 10, 742–761. 10.1039/D1TC04727B.

[ref50] Dualeh A.; Moehl T.; Tétreault N.; Teuscher J.; Gao P.; Nazeeruddin M. K.; Grätzel M. Impedance Spectroscopic Analysis of Lead Iodide Perovskite-Sensitized Solid-State Solar Cells. ACS Nano 2014, 8 (1), 362–373. 10.1021/nn404323g. 24341597

[ref51] Fabregat-Santiago F.; Kulbak M.; Zohar A.; Vallés-Pelarda M.; Hodes G.; Cahen D.; Mora-Seró I. Deleterious Effect of Negative Capacitance on the Performance of Halide Perovskite Solar Cells. ACS Energy Lett. 2017, 2 (9), 2007–2013. 10.1021/acsenergylett.7b00542.

[ref52] Khan M. T.; Huang P.; Almohammedi A.; Kazim S.; Ahmad S. Mechanistic Origin and Unlocking of Negative Capacitance in Perovskites Solar Cells. iScience 2021, 24, 102024 10.1016/j.isci.2020.102024. 33521597 PMC7820557

[ref53] Hernández-Balaguera E.; Bisquert J. Time Transients with Inductive Loop Traces in Metal Halide Perovskites. Adv. Funct. Mater. 2023, 2308678 10.1002/adfm.202308678.

[ref54] Dunbar R. B.; Duck B. C.; Moriarty T.; Anderson K. F.; Duffy N. W.; Fell C. J.; Kim J.; Ho-Baillie A.; Vak D.; Duong T.; et al. How reliable are efficiency measurements of perovskite solar cells? The first inter-comparison, between two accredited and eight non-accredited laboratories. J. Mater. Chem. A 2017, 5, 22542–22558. 10.1039/C7TA05609E.

[ref55] Unger E.; Paramasivam G.; Abate A. Perovskite solar cell performance assessment. J. Physics: Energy 2020, 2, 044002 10.1088/2515-7655/abaec8.

[ref56] Jeong S.-H.; Park J.; Han T.-H.; Zhang F.; Zhu K.; Kim J. S.; Park M.-H.; Reese M. O.; Yoo S.; Lee T.-W. Characterizing the Efficiency of Perovskite Solar Cells and Light-Emitting Diodes. Joule 2020, 4, 1206–1235. 10.1016/j.joule.2020.04.007.

[ref57] Rakocevic L.; Ernst F.; Yimga N. T.; Vashishtha S.; Aernouts T.; Heumueller T.; Brabec C. J.; Gehlhaar R.; Poortmans J. Reliable Performance Comparison of Perovskite Solar Cells Using Optimized Maximum Power Point Tracking. Solar RRL 2019, 3, 1800287 10.1002/solr.201800287.

[ref58] Pellet N.; Giordano F.; Ibrahim Dar M.; Gregori G.; Zakeeruddin S. M.; Maier J.; Grätzel M. Hill climbing hysteresis of perovskite-based solar cells: A maximum power point tracking investigation. Prog. Photovol. 2017, 25, 942–950. 10.1002/pip.2894.

[ref59] Köbler H.; Neubert S.; Jankovec M.; Glažar B.; Haase M.; Hilbert C.; Topič M.; Rech B.; Abate A. High-Throughput Aging System for Parallel Maximum Power Point Tracking of Perovskite Solar Cells. Energy Technol. 2022, 10, 2200234 10.1002/ente.202200234.

