# Supplementary material for: Accelerating the Assessment of Hysteresis in Perovskite Solar Cells
Source: ACS Energy Lett. 2024 Jan 17;9(2):478–86. doi: 10.1021/acsenergylett.3c02779 (PMC10863394; doi:10.1021/acsenergylett.3c02779)
Supplement: Supplementary file 1 — nz3c02779_si_001.pdf [file nz3c02779_si_001.pdf]

Supporting Information

Accelerating the Assessment of Hysteresis in Perovskite Solar Cells

Enrique H. Balaguera,[1,*] Juan Bisquert[2]

[1]Escuela Superior de Ciencias Experimentales y Tecnología (ESCET), Universidad Rey Juan Carlos, 28933 Móstoles, Madrid, Spain

[2]Institute of Advanced Materials (INAM), Universitat Jaume I, 12006 Castelló, Spain

Corresponding author e-mail: enrique.hernandez@urjc.es

1. Theoretical Background

For the interpretation of our experimental transient current results $\Delta j(t)$, we develop eqs 1−3 into the corresponding small perturbation equations[1] that become:

$$\Delta j(t) = C_g \frac{d(\Delta v)}{dt} + \Delta v g_{rec} + j_d + C_s \frac{d(\Delta V_s)}{dt} \tag{S1}$$

$$C_s \frac{d(\Delta V_s)}{dt} = \Delta v g_{ion} - \Delta V_s g_{ion} \tag{S2}$$

$$L_d \frac{d(\Delta j_d)}{dt} = \Delta v - \frac{\Delta j_d}{g_{elect}} \tag{S3}$$

in response to a voltage step $\Delta V(t) = \Delta v$. Note that the form of the circuit elements (g_{rec}, C_s, g_{ion}, L_d, and g_{elect}) and the respective time constants (τ_s and τ_d) has been defined in the manuscript.

Initially, we analyze the impact of the first two channels in eq S1 on the current response. As the characteristic relaxation times that describe bulk and surface processes are well-separated ($\tau_v \ll \tau_s, \tau_d$),[2] we approximate eq S1 at short time scales as:

$$\Delta j_{fast}(t) = C_g \frac{d(\Delta v - \Delta j_{fast}(t) R_s)}{dt} + (\Delta v - \Delta j_{fast}(t) R_s) g_{rec} \tag{S4}$$

where now the measured current is affected by the series resistance effects due to, at the initial instant $t = 0$, the geometric capacitor C_g is discharged (virtual short-circuit) and all the voltage goes to the series resistance, $\Delta j_{fast}(t = 0) = {}^{\Delta v}/_{R_s}$. Note that, at this timescale, we assume that eqs S2 and S3 do not provide dynamism ($j_d, C_s \, {}^{d(\Delta V_s)}/_{dt} = 0$ A) to the current response, being only visible the bulk processes. Rearranging terms, one obtains the following first-order differential equation for the fast component of $\Delta j(t)$:

$$R_s C_g \frac{d\left(\Delta j_{fast}(t)\right)}{dt} = \Delta v g_{rec} - \Delta j_{fast}(t)(1 + R_s g_{rec}) \tag{S5}$$

An integration of the differential eq S5 starting at ${}^{\Delta v}/_{R_s}$ and using the realistic approximation $1 \gg R_s g_{rec}$ gives the evolution of the resulting current $\Delta j(t)$ during the first part of the chronoamperometric experiments:

$$\Delta j_{fast}(t) = \Delta v g_{rec} + \frac{\Delta v}{R_s} e^{-\frac{t}{\tau_v}} \tag{S6}$$

from which one can obtain the charging time constant $\tau_v = R_s C_g$.

On the other hand, we find the evolution of the ΔV_s and Δj_d by integration since the relations between the input Δv and the internal memory variables in eqs S2 and S3 are now described by ordinary, linear differential equations with constant coefficients:

$$\Delta V_s(t) = \Delta v\left(1 - e^{-\frac{t}{C_s/g_{\text{ion}}}}\right) \tag{S7}$$

$$\Delta j_d(t) = \Delta v g_{\text{elect}}\left(1 - e^{-\frac{t}{L_d g_{\text{elect}}}}\right) \tag{S8}$$

for the initial conditions $\Delta V_s(t = 0) = 0$ V and $\Delta j_d(t = 0) = 0$ A. As ΔV_s models the internal voltage across the interfacial capacitance C_s, the corresponding current that flows through this branch can be easily obtained as follows:

$$C_s\frac{\mathrm{d}(\Delta V_s)}{\mathrm{d}t} = \Delta v g_{\text{ion}} e^{-\frac{t}{C_s/g_{\text{ion}}}} \tag{S9}$$

Therefore, the last two channels in eq S1 that represent the slow components of $\Delta j(t)$ determine the behavior of the response at long time scales:

$$\Delta j_{\text{slow}}(t) = \Delta j_d(t) + C_s\frac{\mathrm{d}(\Delta V_s)}{\mathrm{d}t} = \Delta v g_{\text{elect}} + \Delta v(g_{\text{ion}} - g_{\text{elect}})e^{-\frac{t}{\tau_{\text{kin}}}} \tag{S10}$$

by using the property of nearly continuity of the characteristic time constants (eq 4).

From eqs S6 and S10, we obtain a reasonable approximation of the complete transient response $\Delta j(t)$ due to a small perturbation of voltage Δv in stabilized current-voltage responses of perovskite solar cells:

$$\Delta j(t) = \Delta j_{\text{fast}}(t) + \Delta j_{\text{slow}}(t) = \\ = \frac{\Delta v}{R_s}e^{-\frac{t}{\tau_v}} + \Delta v(g_{\text{rec}} + g_{\text{elect}}) + \Delta v(g_{\text{ion}} - g_{\text{elect}})e^{-\frac{t}{\tau_{\text{kin}}}} \tag{S11}$$

that constitutes the set of equations given in the main text (eqs 5−7). In effect, the steady-state current term (eq 6) is found by eqs S6 (first term on the right-hand side) and S8 (forced response). On the other hand, memory traces of eq 5 are determined by the free or transient responses (terms with exponential dependence) of eqs S8 and S9 by using the coupling property of characteristic time constants in metal halide perovskites (eq 4). Finally, the ultrafast charging process of the bulk capacitance is given by the exponential term of eq S6.

2. Frequency-Domain Analysis

We calculate the impedance spectroscopy response of our model for a small sinusoidal perturbation of angular frequency ω.[3] Since eqs 1−3 now represent a linear system, we apply the Laplace transform, ${}^{\mathrm{d}}/_{\mathrm{d}t} \to s$, yielding:

$$\hat{j} = C_g s\hat{V} + \hat{j}_{\text{rec}} + \hat{j}_d + s\hat{Q}_s \tag{S12}$$

$$\tau_s s\hat{V}_s = \hat{V} - \hat{V}_s \tag{S13}$$

$$\tau_d s\hat{j}_d = \hat{j}_{\text{elect}} - \hat{j}_d \tag{S14}$$

where the tildes represent small ac perturbations.

From eqs S13 and S14, we can obtain, for the variable $s = \mathrm{j}\omega$, $\hat{V}_s$ and $\hat{j}_d$:

$$\hat{V}_s = \frac{\hat{V}}{1 + j\omega\tau_s} \tag{S15}$$

$$\hat{j}_d = \frac{\hat{J}_{elect}}{1 + j\omega\tau_d} \tag{S16}$$

that inserted into eq S12, it leads to the following expression for the ac admittance:

$$Y(j\omega) = \frac{\hat{j}}{\hat{V}} = j\omega C_g + g_{rec} + \frac{1}{\frac{1}{g_{ion}} + \frac{1}{j\omega C_s}} + \frac{1}{\frac{1}{g_{elect}} + j\omega L_d} \tag{S17}$$

where the circuit elements have the values in terms of model parameters indicated in the manuscript; $g_{rec} = {}^{\hat{J}_{rec}}/_{\hat{V}}$, $C_s = {}^{\hat{Q}_s}/_{\hat{V}_s}$, $g_{ion} = {}^{C_s}/_{\tau_s}$, $g_{elect} = {}^{\hat{J}_{elect}}/_{\hat{V}}$, and $L_d = {}^{\tau_d}/_{g_{elect}}$.[4] Unifying the slow relaxation times (τ_s and τ_d) in form of τ_{kin},[5,6] eq 10 in the manuscript can be indeed obtained from eq S17.

In the high-frequency region ($\omega \to \infty$), C_s and L_d behave as virtual short- and open-circuits $-{}^{1}/_{j\omega C_s} \to 0$ and $j\omega L_d \to \infty$–, respectively. Therefore, the high-frequency limit of eq S17 is:

$$Y_{HF}(j\omega) = \left[R_s + \frac{1}{(g_{rec} + g_{ion}) + j\omega C_g}\right]^{-1} \tag{S18}$$

forming a capacitive arc in the complex plane via the transition from the high-frequency limit conductance ${}^{1}/_{R_s}$ to $g_{rec} + g_{ion}$ with the following characteristic relaxation time:

$$\tau_{Y_{HF}} = \tau_v = R_s C_g \tag{S19}$$

as the pole of the transfer function with ${}^{1}/_{R_s} \gg g_{rec}, g_{ion}$.[2]

At sufficiently low frequencies, the inductive and slow capacitive branches become "active" and the conductance can be reduced or increased depending on the dominant mechanism at different voltages. Analogously, the low-frequency limit ($\omega \to 0$) of the admittance is given by:

$$Y_{LF}(j\omega) = g_{rec} + \frac{j\omega C_s + g_{elect}}{1 + j\omega\tau_{kin}} \tag{S20}$$

where the characteristic time constant now is:

$$\tau_{Y_{LF}} = \tau_{kin} = \frac{C_s}{g_{ion}} = L_d g_{elect} \tag{S21}$$

because we consider the geometric capacitor fully charged, ${}^{1}/_{j\omega C_g} \to \infty$ (open circuit). Now, the low-frequency arc remains in the first quadrant (low voltages) or enters the fourth quadrant (high bias points) of the complex plane depending on which conductance is higher, g_{ion} or g_{elect}; i.e., the real part of the admittance Y_r decreases or increases from $g_{rec} + g_{ion}$ to $g_{rec} + g_{elect}$ when $g_{ion} > g_{elect}$ or $g_{ion} < g_{elect}$, respectively. The time constants of eqs S19 and S21 are the inverse of the characteristic frequencies in which

the imaginary part of the admittance of the resulting capacitive or inductive loops have maximum magnitudes.

The impedance is the inverse or reciprocal function of the admittance (refer to eq S17), resulting, without negligible resistance effects, in the following expression:

$$Z(\mathrm{j}\omega) = \frac{1}{Y(\mathrm{j}\omega)} = R_\mathrm{s} + \left[\mathrm{j}\omega C_\mathrm{g} + g_\mathrm{rec} + \frac{1}{\frac{1}{g_\mathrm{ion}} + \frac{1}{\mathrm{j}\omega C_\mathrm{s}}} + \frac{1}{\frac{1}{g_\mathrm{elect}} + \mathrm{j}\omega L_\mathrm{d}}\right]^{-1} \quad \text{(S22)}$$

which can be expressed as the following equivalent circuit model:

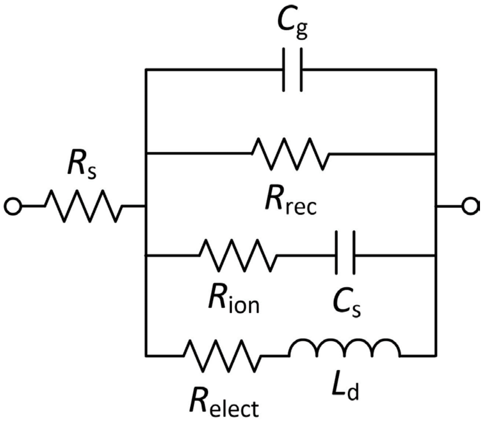

Figure S1. Electrical equivalent circuit obtained by considering an ac small signal excitation in the model.

The equivalent circuit elements are obtained from eq S22 as:

$$R_\mathrm{rec} = \frac{1}{g_\mathrm{rec}} \quad \text{(S23)}$$

$$R_\mathrm{ion} = \frac{\tau_\mathrm{s}}{C_\mathrm{s}} = \frac{1}{g_\mathrm{ion}} \quad \text{(S24)}$$

$$R_\mathrm{elect} = \frac{1}{g_\mathrm{elect}} \quad \text{(S25)}$$

$$L_\mathrm{d} = \frac{\tau_\mathrm{d}}{g_\mathrm{elect}} \quad \text{(S26)}$$

The equivalent circuit model of Fig. S1 is the same as that of reference (6). However, a new physical feature has been introduced here. The two relaxation times of the inductor

$$\frac{L_\mathrm{d}}{R_\mathrm{elect}} = \tau_\mathrm{d} \quad \text{(S27)}$$

and the ionic branch

$$R_\mathrm{ion} C_\mathrm{s} = \tau_\mathrm{s} \quad \text{(S28)}$$

do not coincide. This is because the equations of the slow recombination and surface polarization has been separated into two distinct slow variables in eqs 1−3 of the main text. The new model therefore produces a generalization allowing for two distinct slow processes. In practice, nevertheless, in halide perovskite solar cells the two relaxation times can be unified as:

$$\tau_{\mathrm{kin}} = \tau_{\mathrm{d}} = \tau_{\mathrm{s}} \tag{S29}$$

as remarked after eq 5.

From a similar analysis to that developed in the admittance function, the relaxation times at sufficiently high frequencies for the impedance response results in:

$$\tau_{\mathrm{Z_{HF}}} = \frac{C_{\mathrm{g}}}{g_{\mathrm{rec}} + g_{\mathrm{ion}}} \tag{S30}$$

exhibiting also intermediate processes with their respective characteristic relaxation times typically invisible in the spectral responses, both in admittance and impedance plots. In effect, eq S30 does not correspond to the same relation of circuit elements of that of eq S19. The characteristic low frequency for the impedance spectra $\tau_{\mathrm{Z_{LF}}}$, in this scenario, is practically impossible to estimate accurately due to the overlapping processes and because the equivalent circuit configuration being, in any case, very different from that of eq S21.

To summarize, we show an illustration of the complex plane admittance and impedance plots for a perovskite solar cell at low and high voltages, respectively, in Figure S2a and S2b. The models are given in eqs S17 and S22 and can be obtained from the electrical equivalent circuit of Figure 2a.

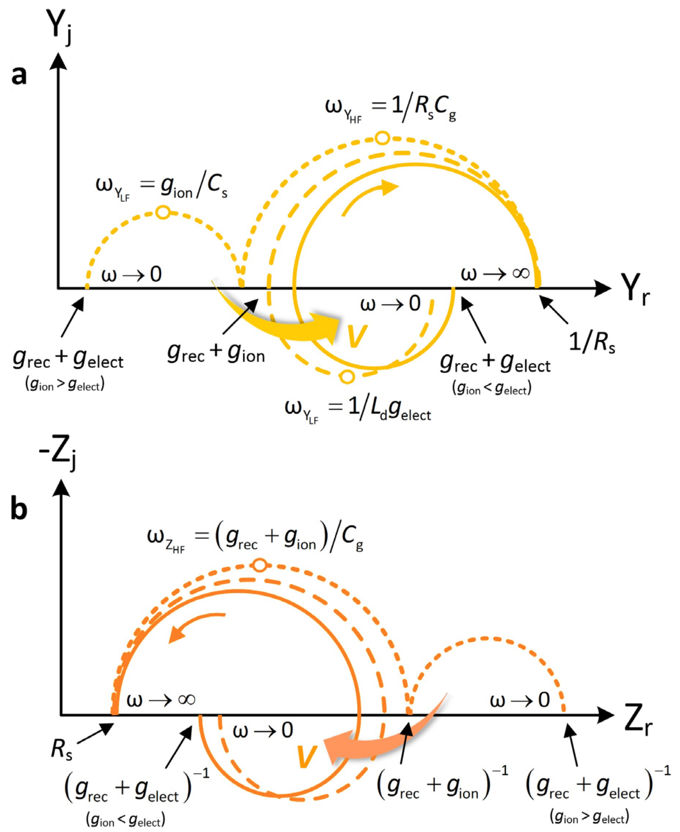

Figure S2. Illustration of the (a) admittance and (b) impedance responses of metal halide perovskite solar cells tracking the transformation from capacitive to inductive behavior in the low-frequency region. Characteristic parameters in form of conductances and time constants are labeled in the spectral patterns.

Further explanations in the correlation of time current transiens and spectral patterns obtained from frequency-resolved techniques (impedance or admittance functions) are given in the reference (7).

3. Impedance of Photovoltaic Perovskites with Regular Configuration

As a guide, Figure S3 shows the impedance forms, extensively reported by many authors, equivalent to the admittance plots shown in Figure 5.

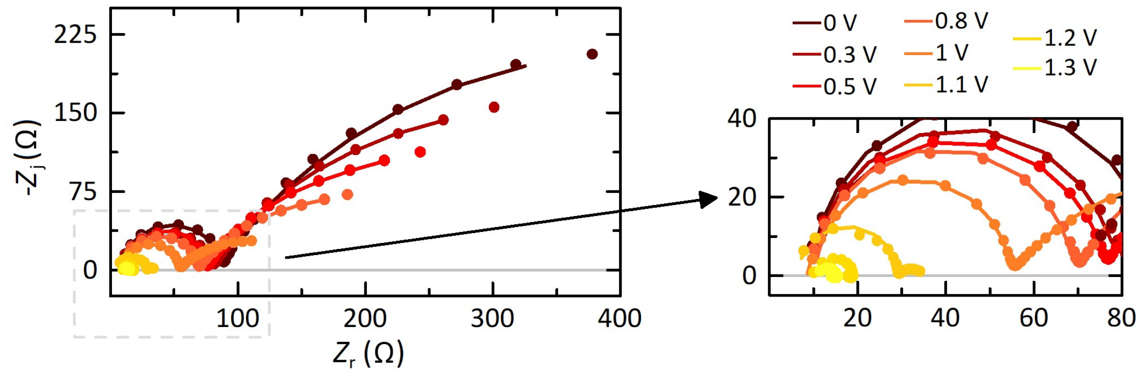

Figure S3. Experimental impedance plots of the quadruple-cation perovskite solar cell with a mesoporous n-i-p (or regular) architecture as a function of the voltage. These spectral patterns correspond to the admittance responses of Figure 5.

4. Control of Hysteresis in Inverted Perovskite Devices

By adapting the control procedure to stabilize current-voltage curves presented here for different device architectures, it is possible to eliminate the hysteresis behavior in all types of perovskite solar cells for a wide variety of experimental situations. For verification purposes, we now developed a consistent analysis of a representative “inverted” or p-i-n configured device with the structure: Tin-doped indium oxide (ITO)/poly[bis(4-phenyl)(2,4,6-trimethylphenyl)amine (PTAA, 6 nm)/perovskite (350 nm)/ phenyl-C61-butyric acid methyl ester (PCBM, 40 nm)/bathocuproine (BCP, 5 nm)/Cu (100 nm) by using the active layer formula $Cs_{0.15}FA_{0.85}Pb(I_{2.85}Br_{0.15})$.

We apply the procedure as follows:

(i) Initially, we develop a preliminary fast current-voltage curve to show the voltage range and the hysteresis level of the perovskite devices (see Figure S4).

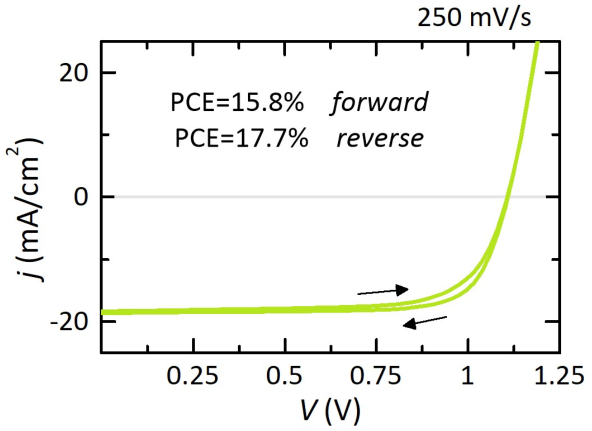

Figure S4. Initial current-voltage experiment to introduce the voltage range and hysteresis of the inverted perovskite solar cell measured under an ultrafast scan rate.

(ii) Then, chronoamperometric measurements are carried out at 0.9 V and 1.2 V, selected as a percentage value of the open-circuit voltage, after applying a voltage step of

10 mV. This value of Δv is fixed to preserve the linearity of the system under study, as performed in Impedance Spectroscopy. Apart from fast capacitive responses of a dielectric origin, time transients decrease and increase in the slow current dynamics, as illustrated in Figure S5a and S5b, respectively. The value of g_{dc} and $g_{ion} - g_{elect}$ are obtained from the final value of the current responses and the difference between the initial point of the slow current dynamics and g_{dc}, respectively (refer to Figure 2b). τ_{kin} is found from the fitting of the transient current response.

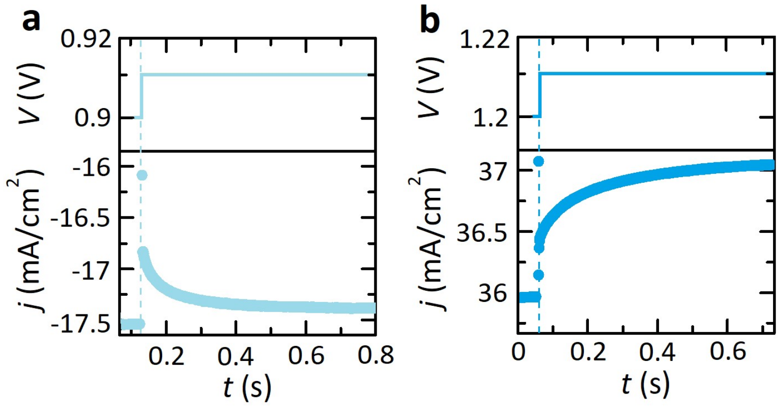

Figure S5. Experimental transient photocurrents in response to a voltage step of 10 mV from different dc voltages (0.9 V in (a) and 1.2 V in (b)) at 100 mW/cm^2.

Alternatively, we will also determine the parameter values of the equivalent circuit of Figure 2a from impedance measurements to obtain, for comparative purposes, Δt_{ss} and s_c as obtained via transient analysis. Figure S6a and S6b show representative examples of experimental admittance and impedance spectra for our inverted device measured at different bias voltages (with and without negative capacitance effects).

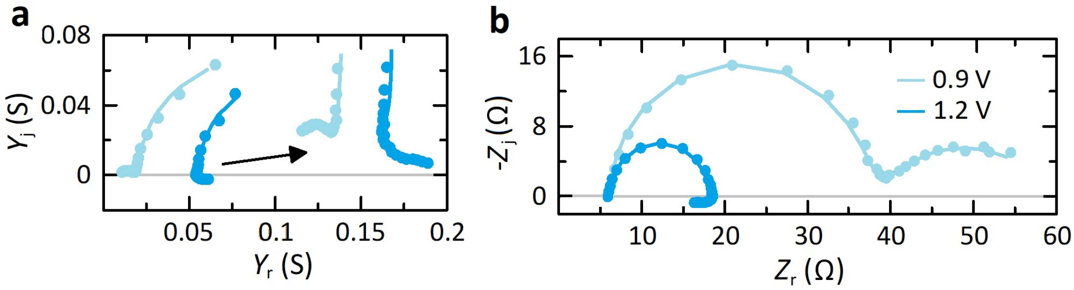

Figure S6. (a) Admittance and (b) impedance plots measured at 0.9 V and 1.2 V bias voltages and under 1 sun equivalent illumination corresponding to the inverted perovskite solar cell under study here.

Here we remark the significant change of response of the solar cell, occurring between the two voltages. In Figure S5 it is observed that the low frequency response is capacitive at 0.9 V and of inductive nature at 1.2 V, as outlined in the representation of Figure S2. Correspondingly, the response to a step voltage at 0.9 V is a simple RC capacitive discharge, while at 1.2 V the current increases with time in the inductive response (refer to Figure S5).

Table 1. Values calculated from the fittings of the transient data shown in Figure S5.

V (V)	$g_{ion} - g_{elect}$ (mS/cm^2)	g_{dc} (mS/cm^2)	τ_{kin} (s)	Δt_{ss} (s)	s_c (mV/s)
0.9	65	10	0.04	0.19	51.31
1.2	-75	100	0.07	0.18	52.66

(iii) Table 1 show the parameters extracted from the fitting of the transient responses that led to the values of the steady-state time and the critical scan rate.

In effect, we now obtain a drastic reduction in the value of s_c as well as in ${g_{ion} - g_{elect}}/{g_{dc}}$ and τ_{kin} in comparison to "regular" or n-i-p perovskite architectures (see Figure 3). The main physical reason behind these results arises from the interface passivation of this type of perovskite devices using organic electron transport materials.[8]

The value of the critical scan rate estimated by using Impedance Spectroscopy remains almost constant as dc voltage increases, resulting also remarkably close to those found from time transient measurements (discrepancies of less than 10%).

(iv) To visualize the effectiveness of our experimental protocol, we obtain current-voltage curves at voltage sweep velocities close to the estimation obtained in (iii), 50 mV/s, to corroborate that this value is the fastest approach that eliminates the hysteresis effects in perovskite solar cells efficiency measurements. Figures S7a indicates that the measured photocurrent exhibits deviations from the operational curve for scan rates superior to 50 mV/s. In contrast, we observe an illuminated current-voltage curve, free of hysteresis influences, in Figure S7b at the scan rate that assure steady-state conditions for all the transient responses throughout the stepwise current-voltage scanning obtained from (ii).

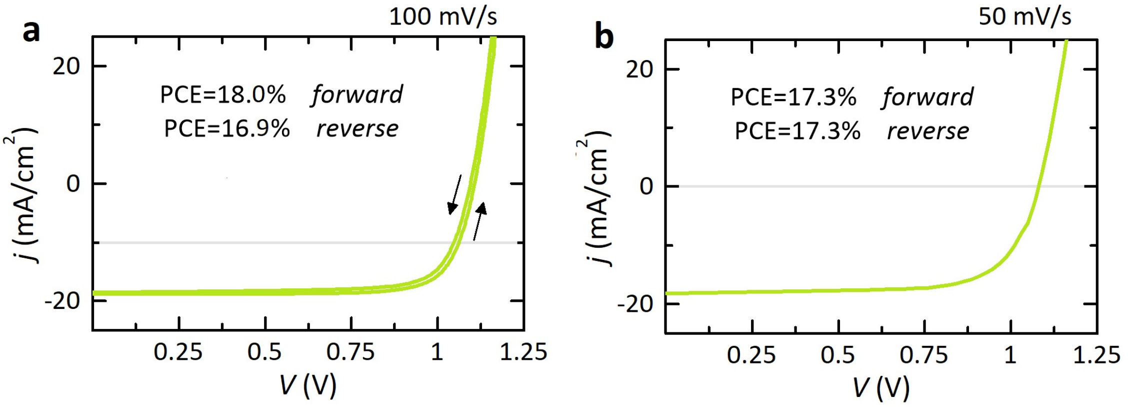

Figure S7. Current-voltage curves under 1-Sun illumination for different scan rates close to the optimal value extracted in (iii): (a) 100 mV/s and (b) 50 mV/s.

From the current-voltage curve measured at 50 mV/s shown in Figure S7b, we calculate the values of the power conversion efficiency in both forward and reverse scans, obtaining a match without casting doubts on the true performance feature of the device (17.3 %) at steady state.

The reproducibility of the experimental data shown in this section was also checked by conducting the chronoamperometric experiments, impedance, and current-voltage measurements in 12 samples with this same architecture. Figures illustrate representative examples of the obtained results and respective simulated data.

5. Experimental Methods

Fabrication of Perovskite Devices with Regular (n-i-p) Architecture. Fluorine-doped tin oxide (FTO) glass sheets were etched with Zn powder and diluted HCl. Then, the etched

substrates were cleaned in an ultrasonic bath with Hellmanex®, acetone, and ethanol for 15 min and finally were immediately dried with dry air. Compact TiO_2 (c-TiO_2) solution was prepared with titanium diisopropoxide bis(acetylacetonate):acetylacetone:ethanol =0.6:0.4:9 (v:v) and then were sprayed onto FTO substrates at 450 °C. Mesoporous TiO_2 (m-TiO_2) paste was prepared with titanium paste:ethanol=1:6 (w:w), and was spin coated to c-TiO_2 substrates at 5000 rpm for 20 s and then annealed at 450 °C for 30 min. After that, the as-prepared FTO/c-TiO_2/m-TiO_2 substrates were quickly transferred to a N_2 filled glove box for perovskite and spiro-OMeTAD deposition. The halide perovskite $Rb_{0.05}Cs_{0.05}MA_{0.15}FA_{0.75}Pb_{1.05}(I_{0.95}Br_{0.05})_3$ was prepared as follows. Briefly, first 1.5 M stock solution of 1) CsI (DMSO), 2) RbI (DMSO), and 3) PbI_2 (DMSO:DMF=1:4) were prepared, respectively. Then 1.5 M 4) $(MABr)_{0.9}(PbI_2)$ (DMSO:DMF=1:4), and 5) $(FAI)_{0.9}(PbI_2)$ (DMSO:DMF=1:4) were freshly prepared by dissolving MABr or FAI power in solution 3), respectively. After that, the solutions were mixed at a ratio of 4):5):1):2):3)=190:950:60:60:60 (v:v) in sequence. The perovskite spin coating process was carried out at 2000 rpm for 10 s, and then 6000 rpm for 30 s. Initially, 50 μL perovskite solution was dropped on a 1.5×2.5 cm^2 FTO/c-TiO_2/m-TiO_2 substrate. During the second step of spin coating, 100 μL chlorobenzene was dropped at 15 s before ending. The samples were annealed at 100 °C for 1 h on a hot plate for crystallization. Hole transporting layer was prepared by dissolving 0.12 g spiro-OMeTAD in 1130 μL chlorobenzene and then doped with 47.3 μL TBP and 23.5 μL Li-TFSI (1.8 M in acetonitrile). The spin coating was conducted at 4000 rpm for 20 s with 50 μL solution. The finished devices were placed inside a dry air box for 12 h to fully oxidize the spiro-OMeTAD. Finally, 80 nm Au was deposited as the front electrode by thermal evaporation. The evaporation rate was controlled in different stages to limit the damage to the spiro-OMeTAD layer.

Fabrication of Inverted Perovskites Solar Cells (p-i-n Configuration). Patterned glass/ ITO substrates were ultrasonically cleaned with soap water, deionized water, and ethanol, followed by UV-ozone treatment for 30 min. All these processes were carried out inside the N_2-filled glovebox with oxygen and moisture levels below 1 ppm. 4 mg/mL PTAA was dissolved in chlorobenzene, and solution spin-coated on glass/ITO substrate at 5500 rpm for 30 s. Then samples are annealed on a hot plate at 100 ºC for 10 min in the N_2-filled glove box. Briefly, dual cation perovskite $Cs_{0.15}FA_{0.85}Pb(I_{2.85}Br_{0.15})$ precursor solutions were deposited from a precursor solution containing FAI, PbI_2, CsI and $PbBr_2$ in anhydrous DMF:DMSO (vol. ratio=9:1). The perovskite solutions were spin-coated in a two-steps program at 2000 rpm for 10 s and 5000 rpm for another 30 s. During the second step, 300 μL of chlorobenzene was poured on the spinning substrate 20 s prior to the end of the program. The perovskite films were further annealed on a hot plate at 100 ºC for 10 min in the N_2-filled glove box. After the perovskite film cooled down to room temperature, a 20 mg/mL PCBM solution in chlorobenzene was spun cast onto the perovskite layer at 1500 rpm for 50 s. Then 1 mg/mL BCP in chlorobenzene solution was deposited onto the PCBM layer at 4000 rpm for 30 s. Finally, the samples were completed by thermally evaporating 100 nm of Cu under the pressure of 10^{-6} mbar through a shadow mask on top of the electron transport layers.

Characterization. Experiments were carried out by using an AutoLab potentiostat/ galvanostat model PGSTAT204 (Eco-Chemie), equipped with the FRA32M impedance module, and a light emitting diode (LED) class AAA solar simulator (Newport/Oriel VeraSol-2). The illumination intensity was adjusted to be equivalent to 100 mW/cm^2 using a calibrated NIST-certified KG5 filtered Si reference cell. The champion solar cells

achieve the following photovoltaic parameters listed below. For the regular device, an open-circuit voltage of 1.11 V, a short-circuit current density of 24.38 mA/cm^2, a fill factor of 76.35%, and power conversion efficiency of 20.64%; for the inverted photovoltaic perovskite (in the same order), 1.11 V, 21.23 mA/cm^2, 75.68%, and 17.83%, both assessed in pristine state, under slow voltage sweep velocities. For experimental current-voltage characterization, samples were initially left at respective polling bias for approximately 10 s. The applied voltage was then swept from the bias voltage up to 1.3 V under illumination, with a 10-mV step at different sweep rates (from 500 to 1 mV/s). Transient photocurrent measurements were obtained by configuring the AutoLab to apply consecutive voltage-steps, in a staircase voltammetry fashion, of 10-mV height and variable delay times as a function of scan rate. A sampling rate of 1 MHz was used to faithfully record the initial-decay region and the subsequent long tail of the photocurrents at long time scales, during stepwise voltage scanning. Finally, frequency-dependent admittance measurements involved the imposition of 10 mV amplitude sinewaves with frequency-values logarithmically spaced between 1 MHz and 1 Hz, under different applied bias. All the experiments at room temperature were performed under ambient atmosphere with the dry-air flow.

References

(1) Gonzales, C.; Guerrero, A.; Bisquert, J. Transition from Capacitive to Inductive Hysteresis: A Neuron-Style Model to Correlate I–V Curves to Impedances of Metal Halide Perovskites. *J. Phys. Chem. C* **2022**, *126*, 13560–13578.

(2) Hernández-Balaguera, E.; Bisquert, J. Negative Transient Spikes in Halide Perovskites. *ACS Energy Lett.* **2022**, *7*, 2602–2610.

(3) Guerrero, A.; Bisquert, J.; Garcia-Belmonte, G. Impedance Spectroscopy of Metal Halide Perovskite Solar Cells from the Perspective of Equivalent Circuits. *Chem. Rev.* **2021**, *121* (23), 14430–14484.

(4) Ghahremanirad, E.; Bou, A.; Olyaee, S.; Bisquert, J. Inductive Loop in the Impedance Response of Perovskite Solar Cells Explained by Surface Polarization Model. *J. Phys. Chem. Lett.* **2017**, *8* (7), 1402–1406.

(5) Ebadi, F.; Taghavinia, N.; Mohammadpour, R.; Hagfeldt, A.; Tress, W. Origin of Apparent Light-Enhanced and Negative Capacitance in Perovskite Solar Cells. *Nat. Commun.* **2019**, *10* (1), 1574.

(6) Bisquert, J. Electrical Charge Coupling Dominates the Hysteresis Effect of Halide Perovskite Devices. *J. Phys. Chem. Lett.* **2023**, *14*, 1014–1021.

(7) Hernández-Balaguera, E.; Bisquert, J. Time Transients with Inductive Loop Traces in Metal Halide Perovskites. *Adv. Funct. Mater.* **2023**, 2308678, DOI: 10.1002/adfm.202308678.

(8) Ravishankar, S.; Gharibzadeh, S.; Roldán-Carmona, C.; Grancini, G.; Lee, Y.; Ralaiarisoa, M.; Asiri, M.; Koch, N.; Bisquert, J.; Nazeeruddin, M. K. Influence of Charge Transport Layers on Open-Circuit Voltage and Hysteresis in Perovskite Solar Cells. *Joule* **2018**, *2*, 788–798.
